# Delivering amoxicillin at the infection site – a rational design through lipid nanoparticles

**DOI:** 10.2147/IJN.S193992

**Published:** 2019-04-23

**Authors:** Daniela Lopes-de-Campos, Rita M Pinto, Sofia A Costa Lima, Tiago Santos, Bruno Sarmento, Cláudia Nunes, Salette Reis

**Affiliations:** 1 LAQV, REQUIMTE, Departamento de Ciências Químicas, Faculdade de Farmácia, Universidade do Porto, Porto, Portugal, shreis@ff.up.pt; 2 INEB – Instituto de Engenharia Biomédica, Universidade do Porto, Porto, Portugal; 3 i3S – Instituto de Investigação e Inovação em Saúde, Universidade do Porto, Porto, Portugal; 4 IINFACTS, Instituto de Investigação e Formação Avançada em Ciências e Tecnologias da Saúde, Instituto Universitário de Ciências da Saúde, Gandra, Portugal

**Keywords:** linolenic acid, dioleoylphosphatidylethanolamine, Box-Behnken design, permeability, mucins, *Helicobacter pylori*

## Abstract

**Purpose:**

Amoxicillin is a commonly used antibiotic, although degraded by the acidic pH of the stomach. This is an important limitation for the treatment of *Helicobacter pylori* infections. The purpose of this work was to encapsulate amoxicillin in lipid nanoparticles, increasing the retention time at the site of infection (gastric mucosa), while protecting the drug from the harsh conditions of the stomach lumen.

**Materials and methods:**

The nanoparticles were produced by the double emulsion technique and optimized by a three-level Box-Behnken design. Tween 80 and linolenic acid were used as potential therapeutic adjuvants and dioleoylphosphatidylethanolamine as a targeting agent to *Helicobacter pylori*. Nanoparticles were characterized regarding their physico-chemical features, their storage stability, and their usability for oral administration (assessment of in vitro release, in vitro cell viability, permeability, and interaction with mucins).

**Results:**

The nanoparticles were stable for at least 6 months at 4°C. In vitro release studies revealed a high resistance to harsh conditions, including acidic pH and physiologic temperature. The nanoparticles have a low cytotoxicity effect in both fibroblasts and gastric cell lines, and they have the potential to be retained at the gastric mucosa.

**Conclusion:**

Overall, the designed formulations present suitable physico-chemical features for being henceforward used by oral administration to treat *Helicobacter pylori* infections.

## Introduction

Amoxicillin (AMX) is a commonly used β-lactam antibiotic that acts by inhibiting the synthesis of bacterial cell walls.^1^ AMX is recommended by international guidelines to be a first-line drug against *Helicobacter pylori (H. pylori)* infections.^2^ These bacteria have been associated with chronic gastritis, peptic ulcers, and cancer.^3^,^4^ Nevertheless, its eradication rates are distant from the desirable for infectious diseases.^2^ The main limitations are caused by the pharmacokinetic properties of antibiotics. For instance, AMX is degraded under the acidic pH of the stomach lumen, mainly due to the hydrolysis of its β-lactam ring.^5^–^7^ The degradation of AMX leads to a need of higher doses and, consequently, to more side-effects.^8^ Our research group have already shown that, at acidic pHs, AMX can promote pore formation in a monolayer of phosphatidylcholines.^9^ The low residence time of AMX in the stomach is also a drawback once *H. pylori* is located at the gastric mucosa.^3^,^10^ Therefore, the treatment of *H. pylori* infections is not feasible in monotherapy.^8^ Instead, two or three antibiotics (eg, amoxicillin, clarithromycin, and metronidazole) are needed, which increases the incidence of side-effects and decreases the therapeutic compliance.^8^

Several micro- and nanoparticles have been suggested as promising strategies to improve the pharmacokinetic properties of antibiotics.^8^,^11^ AMX has being the focus of several studies due to its efficacy. For example, AMX is still effective against *H. pylori*, with generally low resistance rates (1%–3%), contrary to clarithromycin (16%–24%) and metronidazole (20%–40%).^12^ AMX has been encapsulated in several delivery systems,^8^ such as polymeric nanoparticles,^13^–^15^ gastroretentive tablets,^16^,^17^ and liposomes.^18^ However, lipid nanoparticles (LNPs) have not yet been used to encapsulate AMX. LNPs are generally cost-effective and easily scaled-up, which enhance their interest for commercial purposes.^19^,^20^ They are also biocompatible and biodegradable.^19^,^20^ *H. pylori* secrete lipolytic enzymes, including carboxylesterases.^21^,^22^ These enzymes may degrade the lipid matrix, which promotes a local release of the antibiotic. Additionally, Seabra et al^23^ showed the efficacy of LNPs against *H. pylori*. There is also the possibility of functionalizing LNPs to target bacteria and to release the drug near the site of infection.^11^,^24^ There are in fact some targeting options for both *H. pylori* and the gastric mucosa,^25^ that have been used in this work.

**Figure 1:**
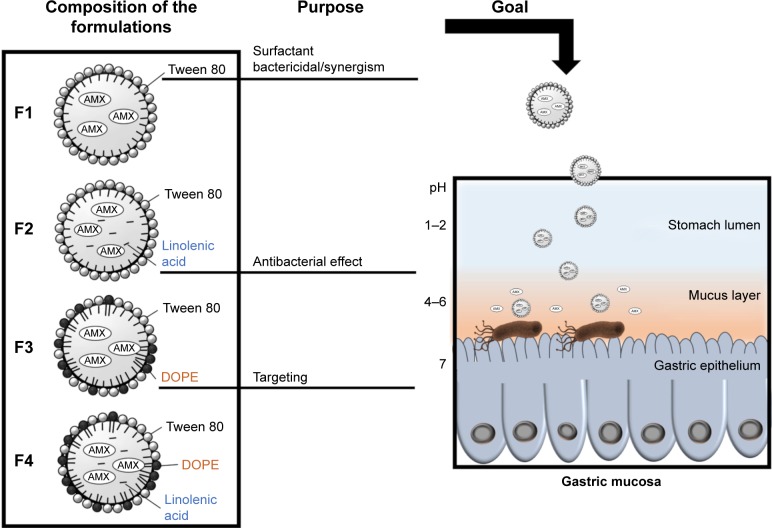
AMX-loaded LNPs, which were designed to release AMX near *H. pylori*. The double-emulsion LNPs are composed of cetyl palmitate, Tween 80, linolenic acid, and DOPE. **Abbreviations:** AMX, amoxicillin; DOPE, dioleoylphosphatidylethanolamine; *H. pylori, Helicobacter pylori*; LNPs, lipid nanoparticles.

The purpose of this study was to design and optimize LNPs to load AMX and target *H. pylori* and to overcome the need of multiple antibiotics by using the antibacterial properties of the LNPs components. Tween 80 was chosen as surfactant due to its ability to detach *H. pylori* outer membrane.^26^ Consequently, it has a synergistic effect with some antibiotics.^26^ Two additional compounds (viz linolenic acid and dioleoylphosphatidylethanolamine [DOPE]) were also added. Four LNPs formulations, which are distinguished by the presence or the absence of the two additional compounds (Figure 1), were studied and characterized. Linolenic acid is an unsaturated fatty acid. These fatty acids are known for their antibacterial properties, their generally low cost, and their abundance in nature.^27^,^28^ Linolenic acid is one of the most potent ones against *H. pylori*, perturbing the integrity of the bacterial membrane.^28^,^29^ Its effect in both coccoid and spiral forms of resistant *H. pylori* was already proven.^30^ On the other hand, DOPE enables an active targeting to *H. pylori* due to the existence of receptors to phospha-tidylethanolamine in these bacteria.^31^ It is in fact with these receptors that these bacteria attach themselves to the antrum of the human stomach.^31^ Thus, DOPE may block the bacterial adhesion to the gastric mucosa, hindering its colonization.^25^

The present work encompasses the full design, development, and characterization of AMX-loaded LNPs for oral administration. By protecting AMX from the acidic pH and combining additional antibacterial strategies, an effective formulation for oral delivery is expected. A Box-Behnken factorial design (BBD) was used to improve the optimization process once it avoids the analysis of one variable at a time, and it evaluates simultaneous interactions among different variables.^32^ Ultimately, it minimizes the costs associated with the optimization process with respect to both time and financial costs.^32^ A comprehensive physical characterization (morphology, particle size, polydispersity [PDI], zeta potential, and loading capacity [LC]) was performed. Moreover, the storage stability, the in vitro release at different pHs, the cytotoxicity in two cell lines, the permeability through gastric cell monolayers, and nanoparticle–mucin interactions were also assessed.

## Materials and methods

### Materials

The lipids Gelucire^®^ 43/01, Gelucire^®^ 44/14, Cetyl palmitate, Compritol^®^ 888 ATO, and Precirol^®^ ATO5 were a kind gift from Gattefossé (Gattefossé, France). Softisan^®^ 100, Dynasan^®^ 116, Imwitor^®^ 900 K, and Imwitor^®^ 491 were gently offered by Sasol (Johannesburg, South Africa). 1,2-di-(9Z-octadecenoyl)-*sn*-glycero-3-phosphoethanol-amine (DOPE) was purchased from Avanti^®^ Polar Lipids (Alabaster, AL, USA). Acetonitrile 99%, chloroform, and acetic glacial acid were obtained from VWR International LLC (Radnor, PA, USA). SIF^®^ Powder was obtained from Biorelevant (Whitechapel, London, UK). Hydrochloric acid was purchased from Fisher Scientific International Inc. (Pittsburgh, PA, USA). Dulbecco’s Modified Eagle’s Medium (DMEM), Roswell Park Memorial Institute (RPMI) medium, trypsin-EDTA (1×), Penicillin (CAS-No 69-57-8)-Streptomycin (CAS-No 3810-74-0), Dulbecco’s Phosphate Buffered Saline 10x pH 7.4 (PBS), Fetal Bovine Serum (FBS), and Hanks’ Balanced Salt Solution (HBSS) with CaCl_2_ and MgCl_2_ were purchased from Gibco^®^ (Invit-rogen Corporation, Paisley, UK). L929 cells (ATCC^®^ CCL-1TM) were acquired from ATCC^®^ (Manassas, VA, USA). MKN-74 cell line was acquired from the BioBank of Instituto de Investigação e Inovação em Saúde (i3S). Amoxicillin trihydrate, linolenic acid, Tween 80, coumarin-6, Thiazolyl Blue Tetrazolium Bromide 98% (MTT), and mucins type II were obtained from Sigma-Aldrich^®^ (St Louis, MO, USA). All components were used without further purification.

## Methods

### Preparation of the AMX-loaded LNPs

Two different methods of synthesis were evaluated, namely, 1) modified free organic-solvent emulsification/sonication method and 2) double emulsion method. A VCX-130 Vibra-Cell™ sonicator, with a CV-18 probe (Sonics & Materials Inc; USA) (130 Watt and 20 kHz) was used in both methods. In the first method, the solid lipid and the surfactant Tween 80 were melted in a hot bath at a temperature above their melting point (from 60°C to 80°C). AMX was solubilized in Milli-Q water using an ultrasounds bath in a frequency of 59 kHz until complete dissolution of the drug. The lipid phase was then dispersed in 6 mL of the pre-heated AMX solution at the same temperature. The emulsion was promoted by sonication for 5 minutes at 70% amplitude and cooled in ice for 30 seconds. In the double emulsion method, the solid lipid and, when applied, the linolenic acid, were dissolved in chloroform (2 mL). AMX was dissolved in a small volume of NaOH (1 M), and Milli-Q water was added until a final volume of 0.5 mL. This solution was then added to the lipid phase. The emulsion was promoted by sonication for 30 seconds at 70% amplitude. When applicable, DOPE (20 mg) was added after dissolution in a small volume of chloroform. A solution of Tween 80 (4 mL, different concentrations) was added to the emulsion, which was then sonicated for 2 minutes at 70% amplitude. A solution of Tween 80 in a lower concentration (4 mL, 12.5 mg/mL) was then added and the resulting LNPs suspensions were placed in a stirring plate at room temperature for 3 hours to evaporate the chloroform. In both methods, the LNPs were protected from light during the entire process of synthesis and storage. Unloaded LNPs were prepared by replacing the drug solution with Milli-Q water. For the permeability studies, coumarin-6 was previously dissolved in a small volume of chloroform, and then it was added to the solid lipid dissolved in the same solvent. The final concentration of coumarin-6 was 0.3% of the total mass of LNPs.

### Experimental design

The BBD was used to optimize and evaluate the effect of different variables on the characteristics of AMX-loaded LNPs.^32^ In this study, a 15-run, 3-factor, 3-level BBD was used, in which the solid lipid mass (X_1_), the concentration of Tween 80 (X_2_), and the AMX mass (X_3_) were defined as the independent variables. The concentration of Tween 80 in the second addition was kept constant (12.5 mg/mL) and only the mass used in the first addition varied. These variables were studied at three different levels (low (-1), medium (0), and high (1), Table S1), which were established from preliminary experiments. The dependent variables were the size (Y_1_), the PDI (Y_2_), and the LC (Y_3_), and the corresponding constraints are also summarized in Table S1. The size and the PDI were selected due to the importance of the diameter of the nanoparticles in gastric diffusion and mucoadhesion.^8^,^33^ The LC was chosen to guarantee that each nanoparticle carries a high amount of drug. The regression analysis for all dependent variables and the response surface plots are also shown in Table S2 and Figure S1, respectively. The experimental runs were generated and further evaluated using STATISTICA Software (v12, StatSoft Inc; Tulsa, OK, USA). The response values that were predicted for the dependent variables were compared with the experimental ones.

### Association efficiency (AE) and loading capacity

The AE was calculated by knowing the amount of AMX encapsulated in the LNPs. The formulations were diluted (1:50) in Milli-Q water and transferred into an Amicon^®^ Ultra-4 Centrifugal Filter Device (50,000 nominal molecular weight limit, NMWL) (MERK Milipore, Ltd; Cork, Ireland). After centrifugation at 524 g until complete separation of the LNPs from the supernatant AMX, the filter unit was centrifuged in an inverted position at 4,713 g during 5 minutes. The pellet of the AMX-loaded LNPs was then dissolved in 2 mL of acetonitrile and centrifuged at 5,251 g until full deposition of the lipid. The entrapped AMX, solubilized in acetonitrile, was quantified using UV-Vis spectroscopy (V-660, Jasco Corporation, Software: Spectra Manager V.2, Jasco Corporation; Easton, MD, USA) at 273 nm. The spectra of the placebos were also measured to ensure that the presence of the other compounds could not interfere with the quantitative measurement of amoxicillin. The AE was calculated as follows:

$$\text{AE}(\%)=\frac{\text{Entrapped AMX amount}}{\text{Total AMX amount}}\times 100$$ (1)

The AE was used to calculate the LC, which was defined by the ratio of the percentage of the drug incorporated into the LNPs and the total amount of lipid in the LNPs:

$$\text{LC}(\%)=\frac{\text{Association efficiency}\times \text{Total AMX amount}}{\text{Total lipid}\;\text{amount}}\times 100.$$ (2)

### Physical characterization of LNPs

Both the size and the PDI were determined in a Particle Size Analyzer by Dynamic Light Scattering (Brookhaven Instruments Corporation; Software: Particle Sizing v.5 Brookhaven Instruments; Holtsville, NY, USA). The results correspond to the mean hydrodynamic diameter (size) and the mean PDI. In the determination of the zeta potential, a Zeta Potential Analyzer (ZetaPALS, Brookhaven Instruments Corporation; Software: PALS Zeta Potential Analyzer v.5 Brookhaven Instruments; Holtsville, NY, USA) was used. The system was operating at a fixed light incidence angle of 90°, at 25°C. The LNPs suspensions were diluted (1:100) in Milli-Q water before the measurements.

Transmission electron microscopy (TEM) was used to assess the morphology of LNPs. LNPs suspensions were diluted (1:4) in Milli-Q water. LNPs suspension (10 µL) were then placed on a copper-mesh grid. After 1–2 minutes, the excess was removed, and 0.75% (w/v) uranyl acetate solution (10 µL) was used for 30 seconds at room temperature to obtain a negative staining. The samples were observed in a JEM-1400 Transmission Electron Microscope (JEOL Ltd., Tokyo, Japan), with an acceleration voltage of 80 kV.

### Assessment of the storage stability

The LNPs in liquid suspension were stored at 4°C for 6 months. The physico-chemical stability was evaluated by measuring the particles size, the PDI, the zeta potential, and the LC over time. All these properties were assessed according to the procedures mentioned in the previous sections. All formulations were protected from light.

### In vitro AMX release study

In vitro release was assessed using the dialysis diffusion technique under sink conditions. A cellulose dialysis bag (Float-A-Lyzer^®^G2 Dialysis Device, 3.5–5 kD MWCO, SpectrumLabs; Rancho Dominguez, CA, USA) was filled with LNPs suspensions (1.5 mL) and placed into a pre-heated (37°C), light protected, and stirred dissolution media (75 mL). The release was assessed in three different pHs to mimic the pH gradient of the gastric mucosa, namely, extremely acidic pH in the stomach lumen, the intermediate pH in the mucus layer, and the pH near to neutral in the epithelial cells (Figure 1).^34^ Thus, this study intended to simulate the release of AMX during the absorption of AMX-loaded LNPs through the gastric mucosa after oral administration. For that purpose, three conditions were defined: 1) 3 hours in fasted state simulated gastric fluid (FaSSGF: NaCl/HCl solution, pH 1.6 with SIF^®^Powder, with bile salts and lecithin); 2) 1 hour in acetate buffer solution (pH 5, *I*=0.13 M), and 3) 22 hours in Hepes buffer solution (pH 7.4, *I*=0.1 M using NaCl). Several aliquots were collected to a UV-Vis microplate (Corning^®^ 96-well UV Microplates, Corning Inc.; Corning, NY, USA) at specific time points and replaced with the same volume of fresh medium. The release rate was quantified by UV-Vis spectroscopy at 277 nm using a microplate reader (BioTek Instruments Inc., Synergy HT, Software: Gen5 v1.08.4, BioTek Instruments Inc.; Winooski, VT, USA). Mathematical models, namely, zero order, first order, Higuchi, and Hixon-Crowell, were fitted to the experimental data to assess the main mechanism of AMX release.^7^ Regression coefficients (r^2^) were used to evaluate the best fitting model.

### In vitro cell viability studies

Cell viability studies were performed using two different cell lines. Mammalian mouse fibroblast cell lines, L929, are recommended by the ISO international standard 10993–5 as a standard cell line for biocompatibility assessment.^35^ MKN-74, a gastric cancer cell line, was used to evaluate any potential gastric cytotoxicity. These cell lines were previously cultured at 37°C in a 5% CO_2_ atmosphere. L929 cells were cultured in DMEM supplied with 10% FBS and 1% Penicillin-Streptomycin, whereas MKN-74 cells were cultured in RPMI culture medium equally supplied with 10% FBS and 1% Penicillin-Streptomycin. A MTT assay was performed to assess cell viability. Briefly, after 80%–90% of confluence, cells were detached from the culture flask by physical detachment using a cell scraper (L929 cell line) or by chemical detachment using 0.25% (w/v) trypsin-EDTA (MKN-74 cell line). The collected cells were seeded in 96-well tissue culture test microplates at a density of 10^5^ cells/well. After adhesion, they were incubated with the LNPs suspensions in different solid lipid concentrations (0.5, 1.0, 2.0, 4.0, and 8.0 mg/mL) for 24 hours (L929 cell line) or for 4 hours (MKN-74 cell line). Different incubation times were used to mimic what may happen in vivo. A longer period of contact with fibroblasts (L929) due to the distribution throughout the human body, and a shorter period for a local interaction with the gastric epithelium (MKN-74). The medium was then replaced by the MTT solution (100 µL, 0.5 mg/mL in fresh medium), and incubated for 2 hours (L929 cells) or 3 hours (MKN-74 cells). Formazan crystals were dissolved in DMSO (100 µL) and its absorbance was measured at 590 and 630 nm using a microplate reader (BioTek Instruments Inc., Synergy HT, Software: Gen5 v1.08.4, BioTek Instruments Inc.; Winooski, VT, USA). The latter was used for background subtraction.

### Permeability studies

Considering that *H. pylori* is located in the interface between the gastric epithelium and the mucus layer of the gastric mucosa,^3^,^10^ it is important to assess the ability of the AMX-loaded LNPs to be retained by the gastric cells. For that purpose, MKN-74 cells (passage number from 5 to 12) were cultured in the same conditions as in the cell viability studies. The cells were seeded on Transwell inserts (polyethylene terephthalate membranes with a pore size of 8 µm, Corning Incorporated, Corning, NY, USA) in a cell density of 1×10^5^ cells per well (growth area of 0.3 cm^2^). After 5 days, the formation of cell monolayers was evaluated using an epithelial VOM2 Voltohmmeter (World Precision Instruments, Sarasota, FL, USA) to measure the transepithelial electric resistance (TEER). The TEER was also measured at the end of the experiment to assess the integrity of the monolayer.

For the permeability studies, 600 and 400 µL of Hank’s salt (HBSS) were used at the basolateral and the apical side, respectively. The study was performed using cell monolayers without and with the addition of porcine mucins type II to mimic the mucus layer. In the cases where mucins were added, the protocol was adapted from elsewhere.^36^ Briefly, 50 µL of mucins at 1% w/v were added to MKN-74 cells in the apical compartment and incubated for 30 minutes at 37°C and at 100 rpm in an orbital shaker. The same volume of HBSS was added to the other cells and incubated in the same conditions. Afterwards, 350 µL of AMX-loaded LNPs labeled with coumarin-6 were added to a final concentration of 2 mg/mL of solid lipid. After 3 hours of incubation, the amount of labelled LNPs in each compartment was determined (λ_exc_ =420 nm and λ_em_ =504 nm). Transwell inserts without cells were used as positive controls, and transwell inserts only with mucins were used to evaluate the ability AMX-loaded LNPs to be retained by mucins.

The percentage of permeability was calculated as follows:

$$\%\text{permeability}=\left[\frac{\left(\frac{F_{\text{basolateral}}}{F_{\text{apical}+\text{basolateral}}}\right)}{\left(\frac{F_{\text{basolateral}\;\text{without cells}}}{F_{\text{apical}+\text{basolateral without cells}}}\right)}\right]\times 100$$ (3)

where F stands for the fluorescence at the maximum of emission (504 nm). The apparent permeability coefficient (P_app_) was calculated according to the following equation:

$$P_{\text{app}}(\text{cm}\cdot s^{-1})=\frac{m{\left(\text{LNPs}\right)}_{\text{normalized}}}{A\times {\left[\text{LNPs}\right]}_{0}\times t}$$ (4)

where m(LNPs)_normalized_ stands for the amount of AMX-loaded LNPs (mg) that permeated the monolayer, normalized by considering the positive control. A is the diffusion area (cm^2^), [LNPs]_0_ is the initial concentration of AMX-loaded LNPs (mg/mL), and t is the time in seconds.

### Nanoparticle–mucin interaction studies

AMX-loaded LNPs were diluted to a final concentration of 2 mg/mL in a solution of HBSS or HBSS with mucins in a concentration of 1% w/v and then incubated for 3 hours at 37°C. The size, the PDI, and the zeta potential were assessed according to the methodology described above.

### Statistical analysis

Statistical analysis was performed using GraphPad Prism Software (v6.01 for Windows; GraphPad Software Inc, San Diego, CA, USA). All assays were performed at least three independent times, and the data are expressed as mean±SD. The data were analyzed using two-way analysis of variance (ANOVA). A *P*-value under 0.05 was considered statistically significant.

## Results and discussion

An initial lipid screening was performed using nine different solid lipids (Gelucire^®^ 43/01, Cetyl palmitate, Compritol^®^ 888 ATO, Precirol^®^ ATO 5, Softisan^®^ 100, Gelucire^®^ 44/14, Dynasan^®^ 116, Imwitor^®^ 900 k, and Imwitor^®^ 491) to prepare LNPs by the modified free organic-solvent emulsification/sonication method. The affinity of AMX to each lipid was evaluated through both the AE and the LC. Furthermore, physical characterization (size, PDI, and zeta potential) was also performed. Cetyl palmitate was selected due to the highest LC, the lower size, and the zeta potential of the LNPs. Furthermore, two different methods of synthesis were evaluated, namely, modified free organic-solvent emulsification/sonication and double emulsion synthesis. Once more, physical characterization, AE, and LC were the key parameters used to evaluate the method of synthesis. AMX-loaded LNPs produced by the double emulsion technique gave the most promising results.

### Experimental design

After the initial screening, preliminary batches of LNPs were used to evaluate the key variables that affected the physical properties and the LC of AMX-loaded LNPs. The key variables, considered as independent variables for the BBD, were the amount of solid lipid, the concentration of Tween 80, and the amount of AMX. Since F4 (see Figure 1) is the most complex formulation, it was selected to be optimized by the experimental design. The mass of both DOPE (20 mg) and linolenic acid (5 mg) was kept constant.

Fifteen formulations suggested by the software were produced and fully characterized. Three out of the 15 formulations corresponded to a central point, which is used to estimate the experimental error. The size, PDI, and LC were selected as dependent variables. The R^2^ values obtained from the correlation between the experimentally measured values and the predicted values were used to evaluate the best fitting model. The quadratic model (equation A.1) was selected due to the higher R^2^ values (Figure S2). A detailed description of the BBD results is provided in the Supplementary materials. An ideal formulation (F4) was suggested by the STATISTICA v12 software (detailed composition in Table 1) by considering the effects of each factor in each dependent variable and the constraints applied for each one. The particle size is an important parameter for gastric diffusion. It is known that particles with >200 nm have a decreased diffusion through the gastric mucosa.^8^ Furthermore, nanoparticles with smaller sizes (50 and 200 nm) showed higher mucoadhesion than particles of 750 nm to the inflamed tissue of gastric ulcers.^33^ On the other hand, the reduction of the size of nanoparticles promotes early drug release in the gastrointestinal tract.^33^,^37^ Thus, Table S1 shows the constraints applied for the size of the LNPs, with low desirability for sizes above 250 nm. For the other dependent variables, the ideal was a minimum PDI and a higher LC.

The produced LNPs had 197 nm, a PDI of 0.137, and a LC of 7.5, which is not statically different from the predicted values (average size of 184 nm, average PDI of 0.103, and an average LC of 7.5). These results validate the experimental design and show the robustness of the model.

### Physico-chemical characterization

After the optimization of the F4 formulation, another three formulations (F1, F2, and F3) were produced (Table 1). The difference among the four formulations was their composition (Figure 1) relative to the presence or absence of linolenic acid and DOPE. The main goal of studying these formulations is to provide insights on the influence of these compounds in the physical properties of the LNPs. Given the usefulness of linolenic acid as an antibacterial adjuvant^30^ and DOPE as an active targeting,^31^ the full characterization of these systems will support future studies of LNPs–*H. pylori* interactions. The respective unloaded LNPs (P1, P2, P3, and P4) were also studied to evaluate the influence of AMX on the physical properties of the LNPs. The composition of each formulation and their physico-chemical characterization are presented in Table 1.

All AMX-loaded LNPs and the corresponding unloaded LNPs were white, milky, and with low viscosity. Furthermore, LNPs aggregation or AMX deposits were not visible in any formulation. The encapsulation of AMX does not significantly affect any of the studied physical properties (*P*>0.05 when comparing the formulation with the respective unloaded LNP). The concentration of Tween 80 may stabilize the aqueous vacuoles, and AMX may be entrapped in those vacuoles and not in the lipid matrix of the LNPs. Thus, the interaction with the lipid phase is minimal, and similar physical properties of the unloaded LNPs and the AMX-loaded LNPs are expected. Moreover, both the PDI and the zeta potential are also similar among all formulations (*P*>0.05). Lower values of particle size distribution (PDI<0.15) and negative zeta potential values (<−35 mV) indicate that all formulations are stable, with low tendency to aggregate.^38^,^39^ These results are coherent with the visible aspect of the formulation.

The amount of Tween 80, linolenic acid, and DOPE affect the size of the LNPs, which are smaller when linolenic acid and/or DOPE are used to replace part of the Tween 80. The difference between the average size among all formulations is statistically different, with the exception of the comparison between F2 and F3. Given the chemical structure of linolenic acid, it is probably entrapped in the lipid matrix. Therefore, it may be establishing that hydrophobic interactions can increase the packing of the lipid molecules. Consequently, it can decrease the size of the LNPs. On the other hand, DOPE may have a stabilizing effect, similar to Tween 80, which reduces even more the interfacial tension between the phases with different lipophilicities.^40^ These results show that, at least partially, DOPE is at the surface of the LNPs. Hence, it can interact with *H. pylori*, fulfilling its purpose of targeting.

The LC also depends on the presence of Tween 80, linolenic acid, and DOPE. The LC of both F2 and F4 formulations are statistically higher than the LC of the other formulations, which indicates that linolenic acid increases the encapsulation of AMX. This is in agreement with the hypothesis of a higher packing of the lipid matrix caused by the linolenic acid, which can promote the formation of larger aqueous vacuoles. Thus, a higher number of AMX molecules can be entrapped. The molecular structure and the effect of both compounds (linolenic acid and DOPE) on the physical properties of the LNPs indicate that these components are within the particles. Furthermore, the low PDI values decrease the hypothesis of micelles formation.

**Figure 2:**
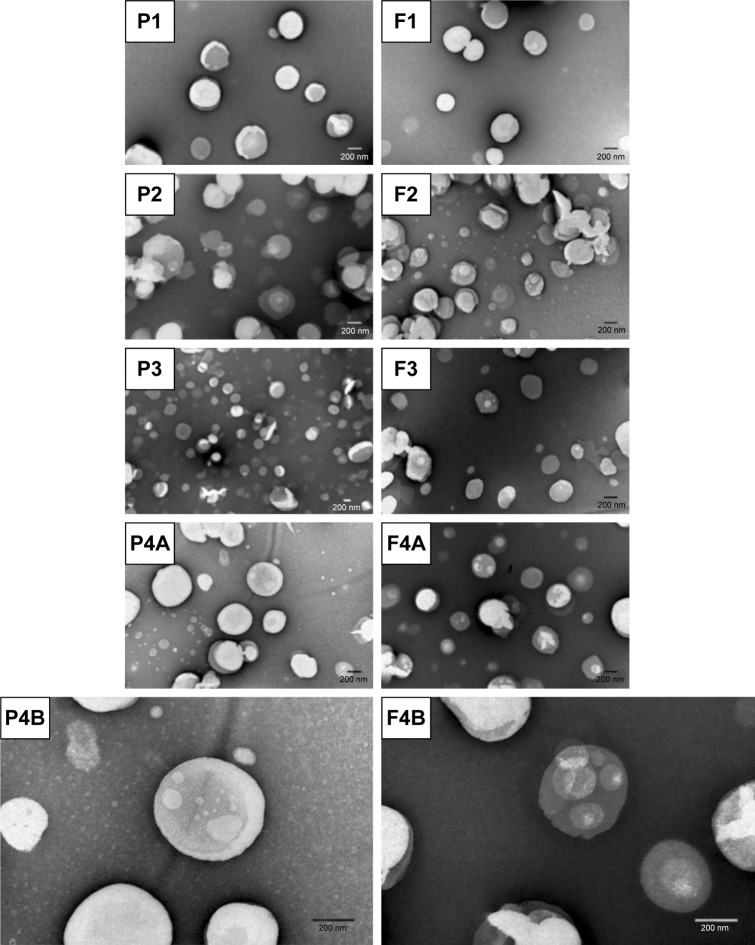
TEM images of the AMX-loaded LNPs and the corresponding unloaded LNPs. **Notes:** P1, F1, P2, F2, F3, P4A, and F4A are at a magnification of 50,000×. P3 is at a magnification of 25,000×. P4B and F4B are at a magnification of 100,000×. **Abbreviations:** AMX, amoxicillin; LNPs, lipid nanoparticles; TEM, transmission electron microscopy.

The morphology of the formulations was also evaluated using TEM (Figure 2). The results revealed that both unloaded and AMX-loaded LNPs have a similar spherical shape with no visible aggregation. This is coherent with the results already determined by dynamic light scattering. TEM images show aqueous vacuoles, which is expected considering the method of the production of the LNPs. In fact, nanoparticles produced by this technique can contain from few to many small compartments, depending on their size.^41^

### Storage stability

**Figure 3:**
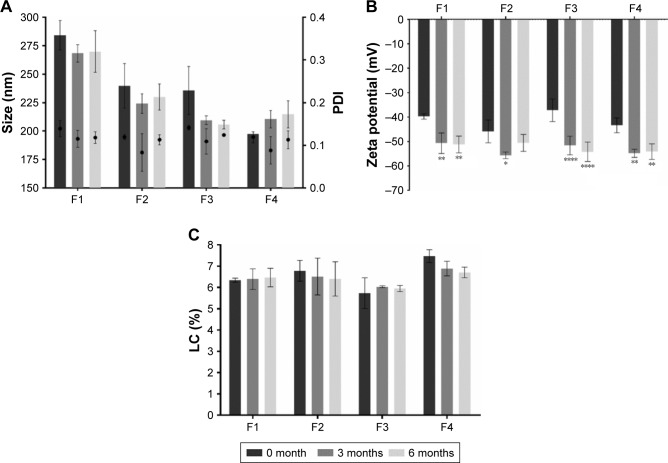
Characterization of the LNPs suspensions. (**A**) LNPs size and PDI. Bars represent the size (left y-axis) and dots represent the PDI (right y-axis). (**B**) LNPs zeta potential. (**C**) LNPs LC. The parameters were evaluated over time (0 months [dark grey], 3 months [intermediate grey] and 6 months [light grey]). **Notes:** Values represent the mean±SD of three independently produced formulations. *P<0.05, **P<0.01, ****P<0.0001 relative to 0 month. **Abbreviations:** LC, loading capacity; LNPs, lipid nanoparticles; PDI, polydispersity.

LNPs suspensions were kept for 6 months at 4°C, protected from the light. The particles size, the PDI, the zeta potential, and the LC were evaluated at weeks 0, 1, and 2, and then every month until the end of the study. Given the amount of data, only the results of 3 months (0, 3, and 6 months) are shown for each property (Figure 3). The detailed results of both unloaded and AMX-loaded LNPs at other time points are presented in Figures S3–S6.

All AMX-loaded LNPs were stable over at least 6 months with respect to both size and PDI (Figure 3A). No statistically significant variations were verified, except for P4 (see Figure S3). All the formulations showed a homogenous size distribution (PDI<0.15) with a mean diameter lower than 300 nm. Furthermore, despite the variances over time in the zeta potential value (Figure 3B), their highly negative values (always <−35 mV) reflect the high stability of the LNPs. These physical characteristics affect several pharmacokinetic and pharmacodynamic properties, such as in vivo distribution and toxicity.^39^ Therefore, their stability is crucial for a drug delivery system.^39^

The stability of the LC over time reflects the ability of a nanoparticle to avoid drug release during storage. High rates of drug release during storage implies that a higher number of nanoparticles must be administered to achieve the same therapeutic effect. Consequently, the costs increase due to the need of higher quantities of matrix materials.^39^ The LC of all AMX-loaded LNPs (Figure 3C) did not significantly change (*P*>0.05) during storage for at least 6 months, which shows a high stability.

### In vitro AMX release study

**Figure 4:**
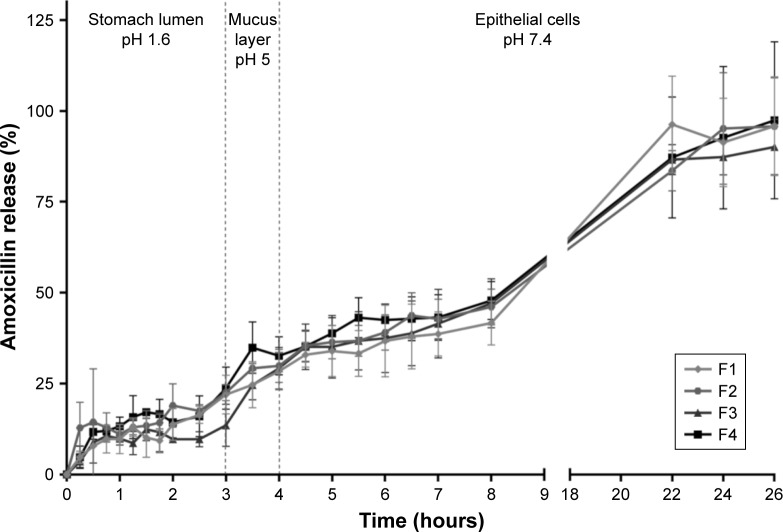
In vitro AMX release profiles from the LNPs in three simulated conditions, namely (i) pH 1.6, (ii) pH 5.0, and (iii) pH 7.4. **Notes:** Vertical lines represent media changes. Values represent the mean±SD of three independently produced formulations. **Abbreviations:** AMX, amoxicillin; LNPs, lipid nanoparticles.

In vitro release studies are usually the simplest method to obtain new insights with respect to the pharmacokinetics of the drug delivery system. The dialysis method was used in different pHs to get closer to the media existent in an absorption through the gastric mucosa, namely, 1) gastric medium (pH 1.6) for 3 hours, 2) mucus layer medium with an intermediate pH (pH 5.0) for 1 hour, and 3) epithelial cells and systemic circulation environment (pH 7.4) until full release. Furthermore, the mimetic gastric medium also contains bile salts and lecithin (FASSGF) to improve the extrapolation. The results obtained from the in vitro release studies of all AMX-loaded LNPs are shown in Figure 4.

The profiles of AMX release are similar for all formulations, which reveal that the presence of linolenic acid and DOPE do not affect the diffusion process of AMX through the LNPs. Moreover, they all followed a Higuchi model release. The release from double emulsions can usually occur in two different ways, namely disintegration of the thin film that stabilizes the aqueous vacuoles or diffusion/permeation processes.^42^ Since the Higuchi model is based on the laws of diffusion, these results show that AMX is released from the LNPs by diffusion. An initial burst release of ~15% occurs within the first hour, which in principle corresponds to the non-encapsulated AMX. This result indicates that the LC may be even higher (around 9.5%) than that measured during LNPs characterization (5.7% to 7.7%). After the initial burst release, all formulations remained stable under the harsh conditions of the gastric medium (pH 1.6) with low rate of AMX release for the first 3 hours. More precisely, <25% was released, which shows that the acidic pH of the gastric medium, the presence of bile salts and lecithin, and the temperature (37°C) do not compromise the integrity of the LNPs. Then a controlled and sustained release was achieved at both pH 5.0 and pH 7.4, with 100% released only after 26 hours.

### In vitro cell viability studies

**Figure 5:**
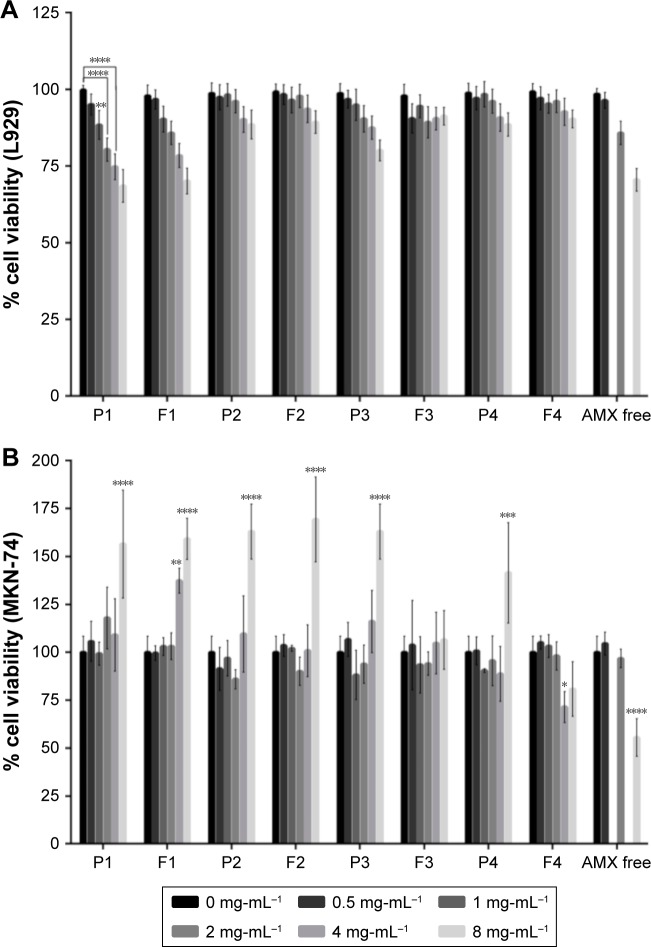
In vitro cell viability studies. (**A**) L929 cell viability study. (**B**) MKN-74 cell viability study. Different formulations in different solid lipid concentrations, from 0 (black) to 8 (light gray) mg/mL of solid lipid were evaluated. For free AMX, the same amount of AMX existent in those concentrations of LNPs was used, with the exception of 1 and 4 mg/mL. **Notes:** Values represent mean±SD of three independently produced formulations. *P<0.05, **P<0.01, ***P<0.005, ****P<0.0001 relative to 0 mg/mL. **Abbreviations:** AMX, amoxicillin; LNPs, lipid nanoparticles.

In vitro cell viability studies are usually the first step to assess the toxicity of drug delivery systems. In this study, two different cell lines were used, namely, fibroblasts (L929) and gastric cells (MKN-74). L929 is recommended by the ISO international standard 10993–5,^35^ and MKN-74 belongs to the first barrier found in vivo when the drug delivery system is administrated by the oral route. The viability of both cell lines was assessed after incubation with LNPs at different solid lipid concentrations or with plain AMX. It is noteworthy that 0.5 mg/mL of solid lipid contain a drug concentration of 0.056 mg/mL, which is higher than the AMX plasmatic concentration (0.0146 mg/mL) and the AMX concentration in the gastric juice (0.00013 mg/mL).^43^ Figure 5 shows the results of the cell viability studies.

The ISO international standard 10993–5 considers that a cytotoxic effect exists when the cell viability is reduced to lower than 70% of the blank.^35^ The L929 viability (Figure 5A) was always higher than 70%, except for 8 mg/mL of solid lipid in both P1 and F1 formulations. In the case of MKN-74 (Figure 5B), the percentage of cell viability increased for all formulations with 8 mg/mL of solid lipid, with the exception of F3 and F4 formulations. There was also a significant increase in the percentage of cell viability when 4 mg/mL of F1 was used. The increased MTT signal can reflect an increase of the proliferation rate or of the mitochondrial activity.^44^ Hence, the formulations with 8 mg/mL of solid lipid can interfere either with the normal growth or with the metabolism of MKN-74. This effect is not verified for both F3 and F4 formulations. In this context, we hypothesize that there is a balance of effects for F3/F4 formulations. The increase of the MTT signal promoted by Tween 80 is balanced by the reduction of the signal promoted by AMX (reduction to 50% of cell viability). This balance does not happen for F1/F2 formulations due to the higher concentration of Tween 80. This theory is also corroborated by the increase of MTT signal on P3 and P4.

The results for both cell lines suggest that the developed AMX-loaded LNPs and the corresponding unloaded LNPs do not interfere with the cell viability until 4 mg/mL of solid lipid, with the exception of F1. These doses are significantly higher than the plasmatic concentration of AMX. Therefore, these LNPs have the potential to be a safe AMX delivery system for oral administration.

### Permeability assays

For the permeability studies, MKN-74 monolayers were used as a human transwell gastric cell model. Before the addition of the LNPs, the TEER values were above 400 Ω⋅cm^2^. A monolayer with tight junctions similar to those that exist in a gastric epithelium present a TEER value superior to the reference value ranging from 150–200 Ω⋅cm^2^. Therefore, the gastric epithelial barrier was successfully produced. The TEER values were also measured after the incubation with the LNPs. The preservation of the TEER values (data not shown) suggests that the tight junctions between MKN-74 cells were still functional and the barrier was intact after the incubation with the LNPs.

The permeability of the LNPs were assessed in four distinct systems: 1) without cells and without mucins, which worked as a positive control; 2) without cells and with mucins to assess the ability of the LNPs to pass through the network of mucins, which are the major component of the mucus layer; 3) with cells and without mucins to evaluate the permeability of LNPs across gastric epithelial cells; and 4) with cells and with mucins to approximate the model to the in vivo gastric mucosa. The results obtained for the percentage of permeability and the apparent permeability coefficient are presented in Table 2. Only AMX-loaded LNPs were evaluated once their physico-chemical properties are similar to the respective unloaded LNPs.

The AMX-loaded LNPs were not able to cross the gastric epithelial cells, even without mucins. Considering that *H. pylori* is mainly attached to the surface of gastric epithelial cells,^45^ this retention may be a huge advantage to increase the efficacy of the system. This is particularly important in the case of AMX. It was already reported that the effect of AMX is not concentration-dependent as other antibiotics.^46^ Instead, it has a time-dependent behavior.^46^ Consequently, a higher retention time near *H. pylori* will increase the efficacy of AMX. In all formulations, roughly 30% of the AMX-loaded LNPs were able to cross the gel-forming mucins. There are no significant differences among the different AMX-loaded LNPs formulations despite the variation of their sizes. This may lead to the conclusion that the retention of the LNPs is driven by a chemical interaction instead of a physical obstruction. Therefore, nanoparticle–mucin interaction studies were performed.

### Nanoparticle–mucin interactions studies

**Figure 6:**
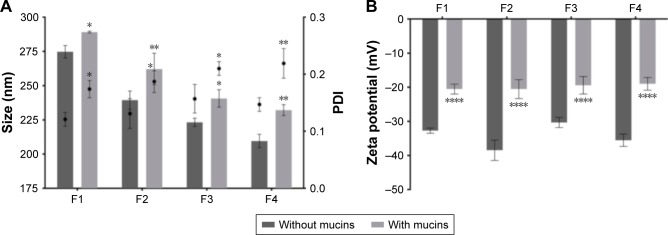
Characterization of the AMX-loaded LNPs suspensions before (dark gray) and after (light gray) the incubation with mucins. (**A**) LNPs size and PDI. Bars represent the size (left y-axis) and dots represent the PDI (right y-axis). (**B**) LNPs zeta potential. **Notes:** Values represent the mean±SD of three independently produced formulations. *P<0.05, **P<0.01, ****P<0.0001 relative to the LNPs without mucins. **Abbreviations:** AMX, amoxicillin; LNPs, lipid nanoparticles; PDI, polydispersity.

In a simplistic way, mucoadhesiveness can be defined as the ability of some compounds to be attracted to a mucous membrane.^25^ In this work, nanoparticle–mucin interaction studies were performed by evaluating changes in the physical properties of the AMX-loaded LNPs after incubation with mucins. The results are presented in Figure 6.

Mucins have surface groups that are negatively charged, such as sialic and sulfonic groups.^47^ Considering the negative charge of the developed LNPs, an electrostatic repulsion would be expected. Interestingly, the incubation with mucins significantly increased the size, the PDI, and the zeta potential of all formulations, which indicates that the AMX-loaded LNPs can interact with mucins. There are other studies that also show the interaction between negatively charged compounds and mucins.^47^–^49^ There are indeed different theories to explain the complexity of the adhesion to the mucus layer, such as electrostatic interactions, hydrogen bonds, van-der-Waals forces, hydrophobic interactions, among others.^25^ Considering the molecular structure of the surface components of the LNPs, Tween 80, and DOPE (Figures S7 and S8, respectively), hydrogen bonds can be established between the LNPs and mucins. For instance, carboxylic groups can interact with sugar residues of the monosaccharide side chains.^48^ Moreover, mucins also have positively charged groups, namely, the charged amino-acids of mucins non-glycosylated domains.^49^ These groups can establish electrostatic interactions with the negatively charged groups of the surface of the LNPs, if the repulsion is masked. This camouflage effect can happen especially in the presence of salts,^47^,^49^ such as the ones present in the HBSS medium (CaCl_2_ and MgCl_2_). Furthermore, the zeta potential of mucins increases as the pH is reduced.^50^,^51^ Therefore, less electrostatic repulsions are expected in vivo at lower pH values.

Besides the *H. pylori* that are attached to epithelial cells, there are bacteria randomly distributed in the mucus layer.^45^ At lower pH values, mucins form a gel.^52^ Nevertheless, *H. pylori* is able to change the rheological properties of the mucus layer due to urease secretion.^53^ By increasing the pH, *H. pylori* reduces the viscosity of the surrounding environment, which can ultimately improve the interactions between the LNPs and mucins. Mucoadhesiveness can in fact be one way of targeting the gastric mucosa, improving the time of contact between the drug and *H. pylori* and, consequently, the efficacy against these bacterial infections.^25^

## Conclusion

In the present work, AMX-loaded LNPs were produced by the double emulsion technique and optimized by a 3-factor, 3-level BBD. As expected by the experimental design, the LNPs have a particle size suitable for diffusion through the mucus layer (around 200 nm).^8^,^33^ Furthermore, these LNPs have physico-chemical features suitable for the purpose, namely, a low PDI, a high LC, and a long storage stability. In vitro release studies revealed that the AMX-loaded LNPs were stable even under harsh conditions (pH 1.6, bile salts, lecithin, and physiological temperature). Since one of the limitations of AMX is its degradation under acidic pH,^6^ the resistance of the nanoparticle to harsh conditions can overcome that limitation. By taking advantage of the lipolytic enzymes secreted by *H. pylori*^21^,^22^ and by using an active targeting (DOPE), a local release near the site of infection can be expected. Furthermore, the AMX-loaded LNPs can be retained near the bacteria by the low permeability through gastric epithelial cells and their ability to interact with mucins. Since the effect of AMX is time-dependent,^46^ a higher retention time near *H. pylori* will increase the efficacy of AMX. Moreover, the development of resistance against these LNPs would be improbable once multiple mutations would be required to fight against different antibacterial compounds (AMX, Tween 80, and linolenic acid).^24^ These results, combined with the low cytotoxic effect in both fibroblasts and gastric cells, reveal that the produced LNPs may have been potential to be administered by oral route against *H. pylori* infections. Studies to evaluate the pharmacodynamic properties of these AMX-loaded LNPs are ongoing.

## Supplementary Materials



## Figures and Tables

**Table 1:** Composition and physical characterization (size, PDI, zeta potential, and LC) of the four AMX-loaded LNPs and the corresponding unloaded LNPs

Formulation	P1	F1	P2	F2	P3	F3	P4	F4

mg								
Cetyl palmitate	178.5	178.5	178.5	178.5	178.5	178.5	178.5	178.5
Tween 80	130	130	125	125	110	110	105	105
Linolenic acid	–	–	5	5	–	–	5	5
DOPE	–	–	–	–	20	20	20	20
AMX	–	19.9	–	19.9	–	19.9	–	19.9

Size (nm)	291±5	284±11	243±17	240±16	237±12	236±18	178±9	197±2
PDI (±0.02)	0.14	0.14	0.12	0.12	0.12	0.14	0.11	0.12
ζ (mV)	-48±1	-40±1	-39±5	-46±4	-42±4	-37±4	-42±2	-43±3
LC (%)	–	6.3±0.1	–	6.8±0.4	–	5.7±0.6	–	7.5±0.2

**Abbreviations:** AMX, amoxicillin; DOPE, dioleoylphosphatidylethanolamine; LC, loading capacity; LNPs, lipid nanoparticles; PDI, polydispersity.

**Table 2:** Percentage of permeability and apparent permeability (P_app_) of the different AMX-loaded LNPs after 3 hours of incubation in different setups: without cells/with mucins; with cells/without mucins; and with cells/with mucins

	%_Permeability_			P_app_ × 10^−5^ (cm/s)		
	Without cells	With cells		Without cells	With cells	
Mucins	X	–	X	X	–	X
F1	30±1	<LOD	<LOD	3.35±0.06	<LOD	<LOD
F2	31±5	<LOD	<LOD	3.4±0.5	<LOD	<LOD
F3	31±5	<LOD	<LOD	3.5±0.6	<LOD	<LOD
F4	31±4	<LOD	<LOD	3.4±0.4	<LOD	<LOD

**Abbreviations:** AMX, amoxicillin; LNPs, lipid nanoparticles; P_app_, apparent permeability; LOD, limit of detection.

## References

[1] (2006). Meyler’s Side Effects of Drugs: The International Encyclopedia of Adverse Drug Reactions and Interactions.

[2] Malfertheiner P, Megraud F, O’Morain CA, European Helicobacter Study Group (2012). Management of *Helicobacter pylori* infection – the Maastricht IV/Florence consensus report. Gut.

[3] Boyanova L, Mitov I, Vladimirov B (2011). Helicobacter pylori.

[4] Mishra S (2013). Is *Helicobacter pylori* good or bad?. Eur J Clin Microbiol Infect Dis.

[5] Tsuji A, Nakashima E, Hamano S, Yamana T (1978). Physicochemical properties of amphoteric beta-lactam antibiotics I: stability, solubility, and dissolution behavior of amino penicillins as a function of pH. J Pharm Sci.

[6] Nägele E, Moritz R (2005). Structure elucidation of degradation products of the antibiotic amoxicillin with ion trap MS(n) and accurate mass determination by ESI TOF. J Am Soc Mass Spectrom.

[7] Barzegar-Jalali M, Adibkia K, Valizadeh H (2008). Kinetic analysis of drug release from nanoparticles. J Pharm Pharm Sci.

[8] Lopes D, Nunes C, Martins MC, Sarmento B, Reis S (2014). Eradication of *Helicobacter pylori*: Past, present and future. J Control Release.

[9] Lopes D, Nunes C, Fontaine P, Sarmento B, Reis S (2017). Proof of pore formation and biophysical perturbations through a 2D amoxicillin-lipid membrane interaction approach. Biochim Biophys Acta Biomembr.

[10] Mobley HLT, Mendz GL, Hazell SL (2001). Helicobacter pylori: Physiology and Genetics.

[11] Huh AJ, Kwon YJ (2011). “Nanoantibiotics”: a new paradigm for treating infectious diseases using nanomaterials in the antibiotics resistantera. J Control Release.

[12] Fallone CA, Chiba N, van Zanten SV (2016). The Toronto Consensus for the Treatment of *Helicobacter pylori* Infection in Adults. Gastroenterology.

[13] Cai J, Huang H, Song W (2015). Preparation and evaluation of lipid polymer nanoparticles for eradicating *H. pylori* biofilm and impairing antibacterial resistance *in vitro*. Int J Pharm.

[14] Jing ZW, Jia YY, Wan N (2016). Design and evaluation of novel pH-sensitive ureido-conjugated chitosan/TPP nanoparticles targeted to *Helicobacter pylori*. Biomaterials.

[15] Arif M, Dong QJ, Raja MA, Zeenat S, Chi Z, Liu CG (2018). Development of novel pH-sensitive thiolated chitosan/PMLA nanoparticles for amoxicil-lin delivery to treat *Helicobacter pylori*. Mater Sci Eng C Mater Biol Appl.

[16] Awasthi R, Kulkarni GT (2013). Development and characterization of amoxicillin loaded floating microballoons for the treatment of *Helicobacter pylori* induced gastric ulcer. Asian J Pharm Sci.

[17] Dey SK, De PK, De A (2016). Floating mucoadhesive alginate beads of amoxicillin trihydrate: A facile approach for *H. pylori* eradication. Int J Biol Macromol.

[18] Singh DY, Prasad NK (2011). Double liposomes mediated dual drug targeting for treatment of *Helicobacter pylori* infections. Pharmazie.

[19] Battaglia L, Gallarate M (2012). Lipid nanoparticles: state of the art, new preparation methods and challenges in drug delivery. Expert Opin Drug Deliv.

[20] Severino P, Andreani T, Macedo AS (2012). Current state-of-art and new trends on lipid nanoparticles (SLN and NLC) for oral drug delivery. J Drug Deliv.

[21] Ruiz C, Falcocchio S, Pastor FIJ, Saso L, Diaz P (2007). Helicobacter pylori EstV: identification, cloning, and characterization of the first lipase isolated from an epsilon-proteobacterium. Appl Environ Microbiol.

[22] Mendz GL, Lim TN, Hazell SL (1993). Fluorinated probes to measure carboxylesterase activities using 19F NMR spectroscopy: application to erythrocytes and *Helicobacter pylori*. Arch Biochem Biophys.

[23] Seabra CL, Nunes C, Gomez-Lazaro M (2017). Docosahexaenoic acid loaded lipid nanoparticles with bactericidal activity against *Helicobacter pylori*. Int J Pharm.

[24] Pelgrift RY, Friedman AJ (2013). Nanotechnology as a therapeutic tool to combat microbial resistance. Adv Drug Deliv Rev.

[25] Lopes D, Nunes C, Martins MCL, Sarmento B, Reis S, Naik J (2015). Targeting strategies for the treatment of Helicobacter pylori infections. Nano based drug delivery Zagreb.

[26] Figura N, Marcolongo R, Cavallo G (2012). Polysorbate 80 and *Helicobacter pylori*: a microbiological and ultrastructural study. BMC Microbiol.

[27] Jackman JA, Yoon BK, Li D, Cho NJ (2016). Nanotechnology formulations for antibacterial free fatty acids and monoglycerides. Molecules.

[28] Sun CQ, O’Connor CJ, Roberton AM (2003). Antibacterial actions of fatty acids and monoglycerides against *Helicobacter pylori*. FEMS Immunol Med Microbiol.

[29] Jung SW, Thamphiwatana S, Zhang L, Obonyo M (2015). Mechanism of antibacterial activity of liposomal linolenic acid against *Helicobacter pylori*. PLoS One.

[30] Obonyo M, Zhang L, Thamphiwatana S, Pornpattananangkul D, Fu V, Zhang L (2012). Antibacterial activities of liposomal linolenic acids against antibiotic-resistant *Helicobacter pylori*. Mol Pharm.

[31] Lingwood CA, Huesca M, Kuksis A, Lingwood CA (1992). The glycerolipid receptor for *Helicobacter pylori* (and exoenzyme S) is phosphatidyl-ethanolamine. Infect Immun.

[32] Singh B, Kapil R, Nandi M, Ahuja N (2011). Developing oral drug delivery systems using formulation by design: Vital precepts, retrospect and prospects. Expert Opin Drug Deliv.

[33] Hassani S, Pellequer Y, Lamprecht A (2009). Selective adhesion of nanoparticles to inflamed tissue in gastric ulcers. Pharm Res.

[34] Allen A, Flemström G (2005). Gastroduodenal mucus bicarbonate barrier: protection against acid and pepsin. Am J Physiol Cell Physiol.

[35] International Standard ISO 10993-5 (2009). Biological Evaluation of Medical Devices–Part 5: Tests for in vitro Cytotoxicity.

[36] García-González L, Yépez-Mulía L, Ganem A (2016). Effect of β-cyclodextrin on the internalization of nanoparticles into intestine epithelial cells. Eur J Pharm Sci.

[37] Polakovic M, Görner T, Gref R, Dellacherie E (1999). Lidocaine loaded biodegradable nanospheres. II. Modelling of drug release. J Control Release.

[38] Honary S, Zahir F (2013). Effect of zeta potential on the properties of nano-drug delivery systems-a review (Part 1). Trop J Pharm Res.

[39] Singh R, Lillard JW (2009). Nanoparticle-based targeted drug delivery. Exp Mol Pathol.

[40] das Neves J, Sarmento B (2015). Precise engineering of dapivirine-loaded nanoparticles for the development of anti-HIV vaginal microbicides. Acta Biomater.

[41] Iqbal M, Zafar N, Fessi H, Elaissari A (2015). Double emulsion solvent evaporation techniques used for drug encapsulation. Int J Pharm.

[42] Pays K, Giermanska-Kahn J, Pouligny B, Bibette J, Leal-Calderon F (2002). Double emulsions: how does release occur?. J Control Release.

[43] Goddard AF, Jessa MJ, Barrett DA (1996). Effect of omeprazole on the distribution of metronidazole, amoxicillin, and clarithromycin in human gastric juice. Gastroenterology.

[44] Zhang X-Y, Ng TK, Breleń ME (2016). Continuous exposure to nonlethal doses of sodium iodate induces retinal pigment epithelial cell dysfunction. Sci Rep.

[45] Hessey SJ, Spencer J, Wyatt JI (1990). Bacterial adhesion and disease activity in *Helicobacter* associated chronic gastritis. Gut.

[46] Yang JC, Lu CW, Lin CJ (2014). Treatment of *Helicobacter pylori* infection: current status and future concepts. World J Gastroenterol.

[47] Feldötö Z, Pettersson T, Dėdinaitė A (2008). Mucin-electrolyte interactions at the solid-liquid interface probed by QCM-D. Langmuir.

[48] Griffiths PC, Occhipinti P, Morris C, Heenan RK, King SM, Gumbleton M (2010). PGSE-NMR and SANS studies of the interaction of model polymer therapeutics with mucin. Biomacromolecules.

[49] Bravo-Osuna I, Noiray M, Briand E (2012). Interfacial interaction between transmembrane ocular mucins and adhesive polymers and dendrimers analyzed by surface plasmon resonance. Pharm Res.

[50] Caicedo JA, Perilla JE (2015). Effect of pH on the rheological response of recongastric mucin. Ingeniería e Investigación.

[51] Lee S, Müller M, Rezwan K, Spencer ND (2005). Porcine gastric mucin (PGM) at the water/poly(dimethylsiloxane) (PDMS) interface: influence of pH and ionic strength on its conformation, adsorption, and aqueous lubrication properties. Langmuir.

[52] Cao X, Bansil R, Bhaskar KR (1999). pH-dependent conformational change of gastric mucin leads to sol-gel transition. Biophys J.

[53] Celli JP, Turner BS, Afdhal NH (2009). Helicobacter pylori moves through mucus by reducing mucin viscoelasticity. Proc Natl Acad Sci.

