# Supplementary material for: Delivering amoxicillin at the infection site – a rational design through lipid nanoparticles
Source: Int J Nanomedicine. 2019 Apr 23;14:2781–95. doi: 10.2147/IJN.S193992 (PMC6488159; doi:10.2147/IJN.S193992)
Supplement: Supplementary file 1 [file IJN-14-2781-193992.pdf]

Supplementary data

Delivering amoxicillin at the infection site – a rational design through lipid nanoparticles

Daniela Lopes-de-Campos[1, #], Rita M. Pinto[1,#], Sofia A. Costa Lima[1], Tiago Santos[2,3], Bruno Sarmento[2,3,4], Cláudia Nunes[1], Salette Reis[1*]

[#]These authors contributed equally to this work

[1]LAQV, REQUIMTE, Departamento de Ciências Químicas, Faculdade de Farmácia, Universidade do Porto, Portugal; [2]INEB - Instituto de Engenharia Biomédica, Universidade do Porto, Rua Alfredo Allen 208, 4200-393 Porto, Portugal; [3]i3S - Instituto de Investigação e Inovação em Saúde, Universidade do Porto, Rua Alfredo Allen 208, 4200-393 Porto, Portugal; [4]IINFACTS, Instituto de Investigação e Formação Avançada em Ciências e Tecnologias da Saúde, Instituto Universitário de Ciências da Saúde, Gandra, Portugal

Correspondence: Salette Reis, shreis@ff.up.pt.

Experimental design

Different models can be used (linear, two-factor interaction and quadratic models) and the most suitable model was selected depending on the correlation between the expected and the observed values. Each model is reflected in an equation, such as Eq. S1 that considers non-linear relationships (non-linear quadratic model).[1]

$$Y = A_0 + A_1X_1 + A_2X_2 + A_3X_3 + A_4X_1X_2 + A_5X_2X_3 + A_6X_1X_3 + A_7X_1^2 + A_8X_2^2 + A_9X_3^2 \quad \text{(S1)}$$

in which Y is the measured response; A_0 is an intercept; A_1 to A_3 are the linear coefficients; A_4 to A_6 are the interaction coefficients; A_7 to A_9 are the squared coefficients; and X_1, X_2, X_3 are the coded levels of independent variables.[1,2] The terms X_1X_2, X_2X_3 and X_1X_3 represent the linear interaction terms, while Xi^2 represents the quadratic term.[1] The predicted R^2 was analysed to evaluate the fitness of the model.

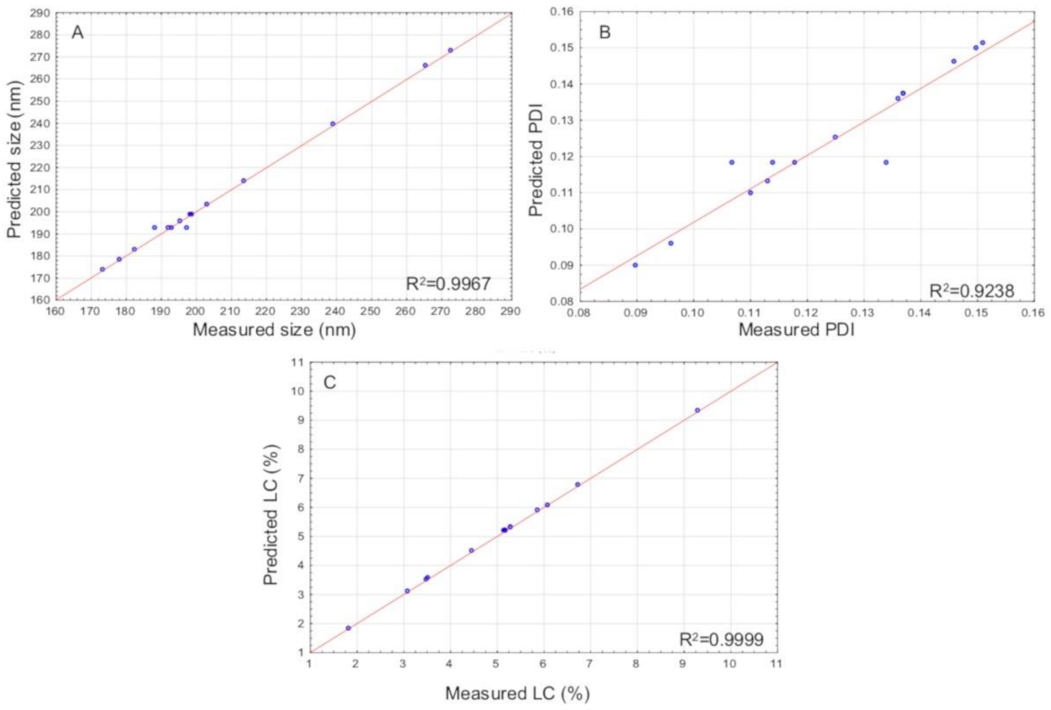

Figure S1 Correlation between the measured and the predicted values of the dependent variables and correspondent R^2 values when fitting with the quadratic model.

Notes: (A) LNPs size. **(B)** LNPs PDI. **(C)** LNPs LC.

Table S1 Independent variables (solid lipid, Tween 80, and AMX mass) and their correspondent levels. Dependent variables (size, PDI, and loading capacity (LC)) and the constraints stablished for the Box-Behnken design.

	**Levels**		
	-1	0	1
**Independent variables**			
X_1 = Cetyl Palmitate mass (mg)	150	200	250
X_2 = Tween 80 mass (mg)	15+50	35+50	55+50
X_3 = AMX mass (mg)	10	20	30
**Dependent variables**	**Desirability**		
	**High**	**Medium**	**Low**
Y_1 = Size	70	150	250
Y_2 = PDI	0	0.1	0.2
Y_3 = LC	20	4	0

The interaction coefficients and the corresponding *p*-values for each dependent variable are shown in Table S2. A synergic effect is represented by a positive sign before the interaction coefficient, which means that the response increases with the increase of the independent variable.

By opposite, a negative sign represents an antagonistic effect. In general, both the size and the LC of the AMX-loaded LNPs depend on the variance of the factors, in terms of both linear and quadratic effects. On the other hand, PDI is not affected by the independent variables ($p>0.05$).

Table S2 Regression analysis for the particle size (Y_1), the PDI (Y_2), and the LC (Y_3), with the correspondent interaction coefficients for the independent variables (solid lipid amount, the concentration of Tween 80, and the amount of AMX).

	**Particle Size**		**PDI**		**LC**	
	**Coefficient**	***p*-value**	**Coefficient**	***p*-value**	**Coefficient**	***p*-value**
**Int**	209.4667	0.000040	0.125750	0.001033	5.01750	0.000001
**Lip (L)**	8.0667	0.041649	0.015917	0.092883	-0.27000	0.000444
**Lip (Q)**	-5.1083	0.049967	0.000896	0.828826	0.17979	0.000488
**Tween 80 (L)**	6.4167	0.063558	-0.004417	0.486725	1.33750	0.000018
**Tween 80 (Q)**	-9.1083	0.016558	-0.000854	0.836570	-0.49146	0.000065
**AMX (L)**	16.7167	0.010182	0.010583	0.179917	1.22250	0.000022
**AMX (Q)**	1.6167	0.306269	-0.005604	0.264096	0.42354	0.000088
**Lip (L) * Tween 80 (L)**	12.8000	0.030308	-0.004750	0.567698	-1.10500	0.000048
**Lip (L) * Tween 80 (Q)**	-12.9500	0.015155	-0.014000	0.105725	0.50250	0.000115
**Lip (Q) * Tween 80 (L)**	-23.2250	0.004786	-0.004000	0.504179	-0.70875	0.000058
**Lip (L) * AMX (L)**	-11.2500	0.038722	-0.004250	0.605787	-0.46000	0.000276
**Lip (Q) * AMX (L)**	-3.7250	0.147141	-0.008500	0.228335	-0.08625	0.003898
**Tween 80 (L) * AMX (L)**	-21.5500	0.011014	0.004250	0.605787	-0.72250	0.000112

Abbreviations: AMX, amoxicillin; L, linear; LC, loading capacity; Lip, solid lipid; PDI, polydispersity index; Q, Quadratic.

Response surface analysis in two dimensions (Figure S2) were calculated from the quadratic polynomial function (Eq. S1). These plots enable a better visualization of the response when different factors are varying.

The particle size of the 15 LNPs formulations was found to be in a range of 173-273 nm. The mean was 209 nm (*Int* value in Table S2). Almost all factors significantly affect the size of the AMX-loaded LNPs. The exceptions are three (Tween 80 (L), AMX (Q), and Lip (Q) * AMX (L)). Tween 80 does not linearly affect the size of the particles. Nevertheless, the quadratic type of interaction has a negative effect (p-value < 0.05). For high amounts of Tween 80, the interfacial tension between phases with different lipophilicities is reduced, which stabilizes smaller particles.[3] The amount of lipid has a positive linear effect, with higher diameters for higher amounts of lipid. Since more lipid molecules are available, the molecular density of the lipid phase in the LNPs will increase. AMX also has a linear positive effect on the size of the AMX-loaded LNPs. This effect shows that, depending on the solid lipid mass and on the T80 concentration, the aqueous vacuoles may not be completely stabilized and AMX may coexist in both lipid and aqueous phase. Simultaneous increase of the lipid

mass and the surfactant concentration led to bigger AMX-loaded LNPs, which is visualized by the positive value of the regression coefficient (12.8000) and the dark red on Figure S2 (top line, left plot). On the opposite, when both the lipid and the AMX mass increase, the LNPs are smaller (negative effect). A similar effect happens with the simultaneous increase of the AMX mass and the Tween 80 concentration.

The PDI varied from 0.09 to 0.15 (mean of 0.126), with no significant effect of all independent variables. The small range of PDI and the lower PDI values show that the selected double-emulsion method promotes the synthesis of a monodisperse suspension.

The LC was found to be in a range of 1.5-9.5 %, with a mean value of 5%. Both linear and quadratic interactions have a statistically significant effect on the LC. The lowest *p*-value is obtained for the linear positive effect of Tween 80. This can be explained by the higher stabilization promoted by Tween 80 of the aqueous vacuoles in which AMX is loaded. AMX also linearly increases the LC once there is a higher amount of drug available for entrapment. On the opposite, higher amounts of lipid decrease the LC once the LC is inversely proportional to the lipid amount (Table S2). The increase of both the lipid mass and the Tween 80 concentration led to a negative effect on the LC, with a regression coefficient of -1.10500 (Table S2) and a light green in Figure S2 (bottom line, left graphic). The increase of both AMX mass and surfactant concentration promoted a small negative effect.

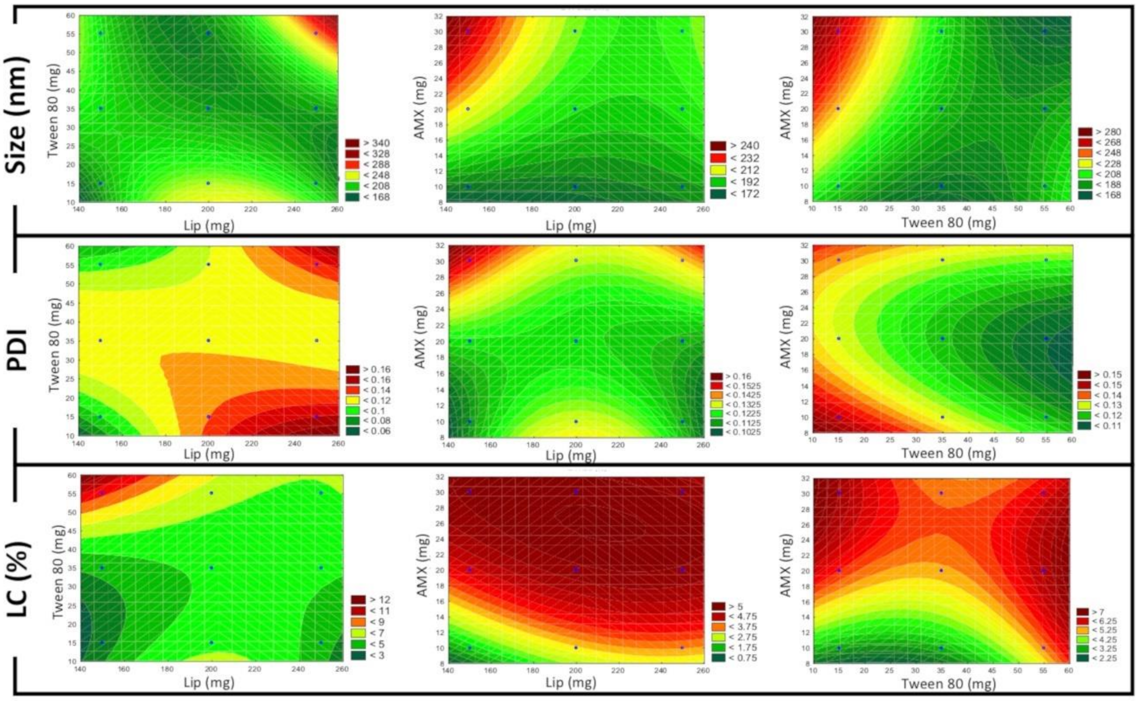

Figure S2 Response surface plots in two dimensions for each dependent variable: size (top line), PDI (middle line), and LC (bottom line). The colours represent the response degree, from green (lowest response level) to dark red (highest response level).

Stability studies

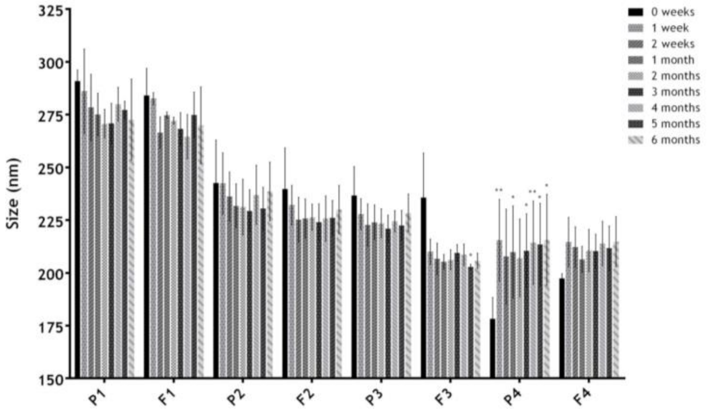

Figure S3 Size of the lipid nanoparticles suspensions (F1 to F4) and respective unloaded nanoparticles (P1 to P4) over time. Values represent the mean ± SD of three independently synthetized formulations. *p < 0.05, **p < 0.01 relatively to 0 months.

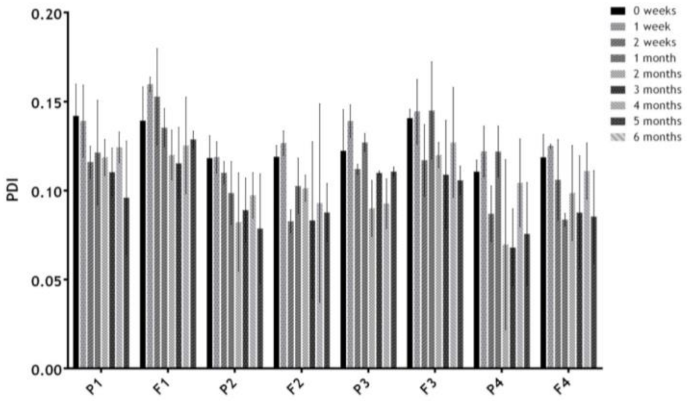

Figure S4 Polydispersion (PDI) of the lipid nanoparticles suspensions (F1 to F4) and respective unloaded nanoparticles (P1 to P4) over time. Values represent the mean ± SD of three independently synthetized formulations.

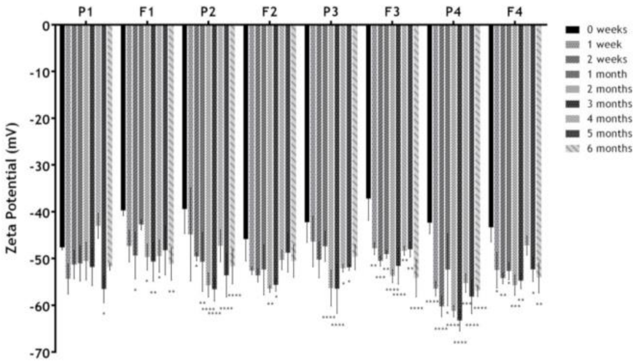

Figure S5 Zeta potential of the lipid nanoparticles suspensions (F1 to F4) and respective unloaded nanoparticles (P1 to P4) over time. Values represent the mean ± SD of three independently synthetized formulations. *p < 0.05, **p < 0.01, ***p < 0.005, ****p < 0.0001 relatively to 0 months.

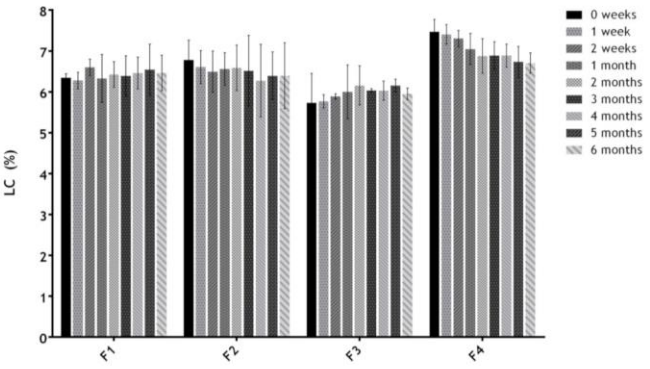

Figure S6 Loading capacity (LC) of the lipid nanoparticles suspensions (F1 to F4) over time. Values represent the mean ± SD of three independently synthetized formulations.

Mucoadhesion studies

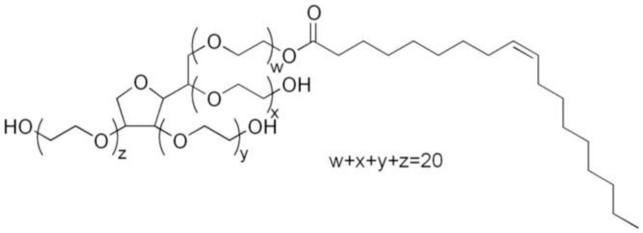

Figure S7 Molecular structure of Tween 80.

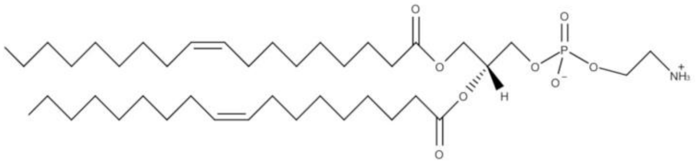

Figure S8 Molecular structure of dioleoylphosphatidylethanolamine (DOPE).

References

1. Lundstedt T, Seifert E, Abramo L, et al. Experimental design and optimization. *Chemometr Intell Lab Syst.* 1998;42:3-40.
2. Hao J, Fang X, Zhou Y, et al. Development and optimization of solid lipid nanoparticle formulation for ophthalmic delivery of chloramphenicol using a Box-Behnken design. *Int J Nanomedicine.* 2011;6:683-692.
3. das Neves J, Sarmento B. Precise engineering of dapivirine-loaded nanoparticles for the development of anti-HIV vaginal microbicides. *Acta Biomater.* 2015;18:77-87.
